# Colchicine to reduce coronavirus disease-19-related inflammation and cardiovascular complications in high-risk patients post-acute infection with SARS-COV-2—a study protocol for a randomized controlled trial

**DOI:** 10.1186/s13063-024-08205-7

**Published:** 2024-06-12

**Authors:** Shani S. Thankachen, Niveditha Devasenapathy, Abhinav Bassi, Arpita Ghosh, Sumaiya Arfin, Balaji Gummidi, Aneesh Basheer, Ashfak Bangi, Dibakar Sahu, Ashish Bhalla, Merlin Blesson, Manish Jain, Vivekanand Jha

**Affiliations:** 1 The George Institute for Global Health India, UNSW, New Delhi, India; 2 Department of General Medicine, Dr. Moopen’s Medical College, Wayanad, India; 3 Department of General Medicine, Jivenrekha Multispeciality Hospital, Pune, India; 4 Department of Pulmonary Medicine, Sleep and Critical Care, All India Institute of Medical Science, Raipur, India; 5 Department of Internal Medicine, Postgraduate Institute of Medical Education & Research, Chandigarh, India; 6 Department of General Medicine, Amrita Institute of Medical Science, Kochi, India; 7 Department of Pulmonary Medicine, Maharaja Agrasen Hospital, Jaipur, India; 8 School of Public Health, Imperial College London, London, UK; 9 Prasanna School of Public Health, Manipal Academy of Higher Education, Manipal, India

**Keywords:** Colchicine, Inflammation, Long COVID, Lung function, Randomized controlled trial

## Abstract

**Background:**

There is no known effective pharmacological therapy for long COVID, which is characterized by wide-ranging, multisystemic, fluctuating, or relapsing symptoms in a large proportion of survivors of acute COVID. This randomized controlled trial aims to assess the safety and efficacy of an anti-inflammatory agent colchicine, to reduce symptoms among those at high risk of developing long COVID.

**Methods:**

This multi-centre, parallel arm, 1:1 individual randomized, placebo-controlled, double-blind superiority trial will enrol 350 individuals with persistent post-COVID symptoms. Participants will be randomized to either colchicine 0.5 mg once daily (< 70 kg) or twice daily (≥ 70 kg) or matched placebo for 26 weeks and will be followed up until 52 weeks after randomization. The primary trial objective is to demonstrate the superiority of colchicine over a placebo in improving distance walked in 6 min at 52 weeks from baseline. The secondary objectives are to assess the efficacy of colchicine compared to placebo with respect to lung function, inflammatory markers, constitutional symptoms, and mental health state. In a sub-sample of 100 participants, cardiac biomarkers of myocardial injury and myocardial oedema using MRI will be compared.

**Discussion:**

Persistent inflammatory response following SARS-CoV-19 is one of the postulated pathophysiological mechanisms of long COVID. Colchicine, a low-cost anti-inflammatory agent, acts via multiple inflammatory pathways and has an established safety profile. This trial will generate evidence for an important health priority that can rapidly translate into practice.

**Trial registration:**

This clinical trial has been registered prospectively on www.clinicaltrials.gov with registration CTRI/2021/11/038234 dated November 24, 2021.

## Introduction

### Background and rationale {6a}

Over 768 million cases of coronavirus disease (COVID-19) have been recorded worldwide since the onset of the pandemic, with 6.95 million deaths [1]. Almost 10% of people infected with the severe acute respiratory syndrome coronavirus 2 (SARS-CoV-2) continued to experience physical, psychological, or cognitive symptoms, even after recovery from the acute infection [2]. Termed “long COVID” [3] or “post-acute COVID syndrome”, this condition is defined by the World Health Organization (WHO) as “…a history of probable or confirmed SARS-CoV-2 infection, usually three months from the onset of COVID-19 with symptoms that last for at least two months and that cannot be explained by an alternative diagnosis” [3]. The most recent estimate of people living globally with long COVID has surpassed 65 million [2]. Without clear preventive and treatment options, this number is steadily increasing [2].

Individuals with long COVID present with common symptoms that include fatigue, malaise, dyspnoea, shortness of breath, and cognitive dysfunction, with an impact on everyday functioning. These symptoms may be new or a continuation of those experienced during the acute illness. The exact mechanism behind the persistence of symptoms is still unknown and likely represents a heterogeneous set of pathophysiological processes. Persistent viral reservoirs, autoimmunity, or direct differential organ injury [4] have been hypothesised as possible reasons for persistent multi-organ symptoms. Continued elevated inflammatory markers point towards persistence of inflammation [5]. Cardiovascular involvement is common, and a downregulation of the respiratory and myocardial angiotensin-converting enzyme 2 (ACE2) pathways, resulting in lung oedema, myocardial inflammation, and acute lung injury have been reported [6]. Additionally, it has been suggested that pro-inflammatory cytokines are upregulated, leading to systemic inflammatory response and multi-organ involvement [7].

Several pharmacological and non-pharmacological treatments are under evaluation for long COVID [8]. The present therapeutic approaches are predominantly rehabilitative in nature, including olfactory training for anosmia and pulmonary rehabilitation for those with respiratory symptoms [9]. There are no proven specific pharmacological treatments to prevent or ameliorate protracted symptoms, making this an important unmet global health need [10].

Colchicine is a well-known anti-inflammatory agent used in rheumatological conditions [11] and recurrent pericarditis [12] with a known safety profile. It blocks nucleotide-binding oligomerization domain-, leucine-rich repeat-, and pyrin domain-containing protein 3 (NLRP3) inflammasome activation driven by SARS-CoV-2 with subsequent downstream activation of pro-inflammatory cytokines interleukin-1 beta (IL-1β) and interleukin-6 (IL-6) which are activated by SARS-Cov2. Colchicine is also an antithrombotic and may attenuate the coagulopathy noted in COVID-19. Of note, a correlation between increasing D-dimer levels and persistent symptoms post-acute infection has been demonstrated [13]. Colchicine has been tried in acute COVID but did not show any benefit. At the time of writing this protocol, no trials were registered evaluating safety and effectiveness of colchicine for prevention and/or treatment of long COVID. We hypothesised that colchicine could improve the functional outcomes in individuals with persistent symptoms following recovery after acute infection with SARS-CoV-2.

### Objectives {7}

The trial will compare treatment with oral colchicine versus placebo for 26 weeks among individuals with clinically confirmed SARS-CoV-2 infection of more than 3 weeks and having persistent symptoms. The primary trial objective is to demonstrate superiority of colchicine for mean difference in change from baseline in 6-min walk test (6MWT) at 52 weeks. The treatment effect of primary interest includes those who discontinued medication for any reason and initiated any other therapy, is in the hypothetical setting where participants do not die, and is in the subpopulation of participants who would begin treatment, regardless of which arm they were assigned to.

The secondary objectives are to assess the efficacy of colchicine compared to placebo with respect to lung function, inflammatory markers, constitutional symptoms, mental health state, and markers of myocardial injury.

### Trial design {8}

**Fig. 1:**
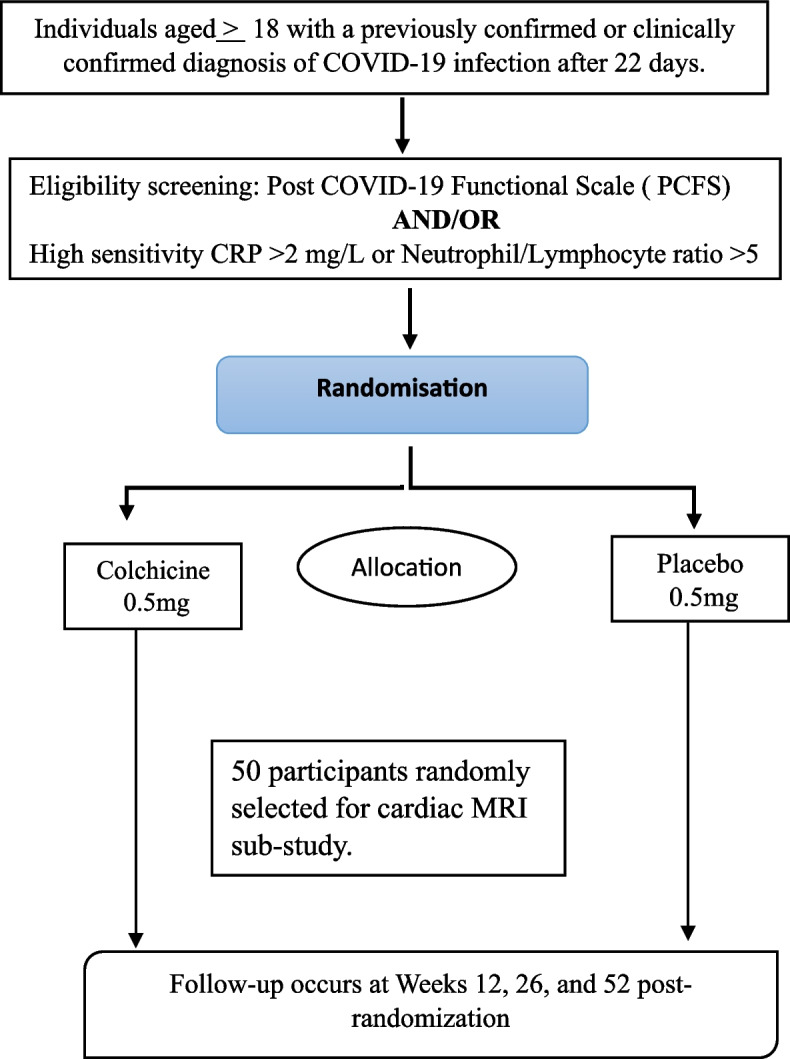
Colchicine study overview

This is a multi-centre, parallel arm, 1:1 individually randomized, placebo-controlled, double-blind superiority trial involving 350 individuals with persistent post-COVID symptoms. Participants will receive either colchicine or a matched placebo for 26 weeks and will be followed up until 52 weeks after randomization. Out of these, 50 participants from each group will be randomly selected for a cardiac MRI sub-study. Figure 1 presents the overview of the study design. This trial is registered with the Clinical Trial Registry of India (CTRI/2021/11/038234), and ethical approvals of the protocol and recruitment materials will be sought from the independent ethics committee at the George Institute for Global Health (ECR/272/Indt/DL/2017/RR-19) and all participating centres before participant recruitment.

## Methods: participants, interventions, and outcomes

### Study setting {9}

This study is being conducting at 5 private and 2 public hospitals in India and one community outreach centre. Sites are selected based on the availability of a list of individuals with COVID-19 and caseload of COVID-19 infection. Participants will be screened, and eligible participants will be recruited.

### Eligibility criteria {10}

Individuals with a history of laboratory or clinically confirmed SARS-COV-2 infection with functional limitation or elevated inflammatory markers will be eligible to participate in the trial. We will use the self-reported flow chart or questionnaire of the Post-COVID-19 Functional Status (PCFS) scale [14] for functional assessment. It is 6-point ordinal scale, ranging from 0 (no functional limitation) to 5 (death). Blood samples for high-sensitive C-reactive protein (HS-CRP) and leucocyte count (total and differential) will be drawn to ascertain eligibility. Informed consent for participation in the trial will be obtained from those found to be eligible as per these criteria by a designated member of the research team.

Inclusion criteria.

1. Age ≥ 18 years
2. Clinically confirmed COVID-19, 22 days since diagnosis prior to the date of randomization
3. Presence of either
4. Post-COVID-19 Functional Status (PCFS) grade 2 or more (higher number indicates poorer status)

-or-

b) Elevated inflammatory markers above normal range high sensitivity CRP > 2 mg/L

-or-neutrophil/lymphocyte ratio > 5

Exclusion criteria.

1. Definite indication for colchicine, such as gout or pericarditis
2. Any contraindication to colchicine, including hypersensitivity to colchicine, pregnant, breastfeeding or of childbearing age and not using an acceptable method of contraception, and currently taking or might need during the trial, a concomitant treatment which is contraindicated with colchicine: cyclosporin, strong CYP3A4 inhibitors, phenylbutazone, immunosuppressants, and anti-neoplastic agents
3. Current/history of inflammatory bowel disease, chronic diarrhoea, blood dyscrasias, or eGFR < 15 mL/min/1.73 m.^2^
4. Current surgical or medical condition that might significantly alter the absorption, distribution, metabolism, or excretion of trial drugs such as prior major gastrointestinal tract surgery (e.g. gastrectomy, lap band, or bowel resection)
5. History of alcohol or drug dependence within 12 months
6. Significant cognitive impairment precluding consent

### Who will take informed consent? {26a}

The information sheets and patient consent forms in English were translated into the regional languages. The information sheet included information on the research purpose, trial procedures, medication safety, risks and benefits of participation, and participant rights and responsibilities. The investigator or the research team will provide information about the proposed trial to all participants, with ample time to read the information sheet thoroughly. Participants can take it back home to discuss with family and are encouraged to discuss with the primary investigator or the research team. The primary investigator or the designated staff will obtain the signature on written consent from the patients interested in participating in the trial.

### Additional consent provisions for collection and use of participant data and biological specimens {26b}

This is not applicable as there is no collection and use of participant data and biological specimens for ancillary studies.

## Interventions

### Explanation for the choice of comparators {6b}

There are no known pharmacological preventive or therapeutic options for symptom relief due to long COVID [9]. Hence, the use of placebo is justified.

### Intervention description {11a}

Eligible and consenting patients will receive either colchicine or matching placebo tablets. The trial intervention is one tablet (0.5 mg) daily for individuals with body weight ≤ 70 kg or one tablet (0.5 mg) twice daily for individuals > 70 kg daily for 26 weeks starting from the day of randomization. The study drug and placebo were manufactured as a single batch and packaged by Steadfast MediSheild Pvt. Ltd in non-transparent sealed bottles containing 50 tablets each.

### Criteria for discontinuing or modifying allocated interventions {11b}

If participant experience any side-effects due to the trial medication, modification of the dose and dosage or withdrawal of the trial medication will be permitted at the discretion of the treating physician. Any modification in dosage and reasons for modification, temporary discontinuation, or permanent discontinuations will be documented in the trial case report forms.

### Strategies to improve adherence to interventions {11c}

The research staff at the clinical site counsel the trial participant at the time of enrolment, monthly telephone check-ins, and during hospital visits on the importance of complying to the advised dose and dosage and to contact the trial staff if they experience any problem. Pill count of the returned tablets will serve as a measure of compliance.

### Relevant concomitant care permitted or prohibited during the trial {11d}

There will be no restrictions to any concomitant therapy during the duration of the trial. All trial participants will be followed up until 52 weeks, regardless of the continuation/compliance to the trial medication, unless the participant withdraws consent.

### Provisions for post-trial care {30}

The study participants are covered by an insurance policy that would cover any unintended non-negligent harm due to trial participation. The proposed treatment regimen in this trial is only 26 weeks. Hence, post-trial access to colchicine tablets to trial participants will not be required even if the study demonstrates evidence of effectiveness compared to placebo.

### Outcomes {12}

We are evaluating several outcomes that measure functional capacity, general and mental health status, respiratory function, inflammatory markers, constitutional symptoms, and cardiovascular outcomes. The primary outcome is the mean difference in change in distance in metres walked in 6 min from baseline at 52 weeks. Table 1 summarizes the trial outcomes and the time points of assessment.

**Table 1:** Trial outcomes

Primary outcome		Time points
Functional capacity	Mean difference in change in distance in metres walked in a 6-min walk test from baseline at 52 weeks (6MWT) [15]	Weeks 12, 26, and 52
Secondary outcomes		
General and mental health	Mean difference in change in Euro-Quality of life (EQoL-5D-5L) [16] score Mean difference in change in Generalized Anxiety Disorder-7 (GAD-7) score [17] Mean difference in change in Patient Health Questionnaire-9 [18] (PHQ-9) depression score	Weeks 12, 26, and 52
Respiratory	Mean difference in dyspnoea score (Borg dyspnoea scale) [19] after completion of 6MWT Mean difference in maximal desaturation during 6MWT Mean difference in change in pulmonary forced vital capacity (FVC) measured using a portable spirometer	Weeks 12, 26, and 52 Weeks 26 and 52
Inflammatory markers	Mean difference in change in high- sensitive C-reactive protein	Weeks 12, 26, and 52
Constitutional symptoms	Mean difference in change in fatigue measured using Chalder Fatigue Scale [20] Median difference in change in self-reported symptom count (symptoms include cough, fatigue, shortness of breath, headache, sore throat, muscle and body aches, reduced appetite, chest pain, palpitations, dizziness, breathlessness, and loss of taste/smell)	Weeks 12, 26, and 52
Sub-study		
Cardiovascular outcomes^a^	Mean difference in change in the high-sensitive troponin T from baseline Mean difference in change in NT-pro BNP Mean difference in change in the following from baseline to week 26 ▪ Degree of myocardial oedema ▪ Late gadolinium enhancement/myocardium ratio ▪ Cardiac volumes ▪ Ejection fraction on cardiac MRI	Week 26
Safety outcomes	Self-reported adverse symptoms	Weeks 12, 26, and 52

^a^Other clinical outcomes will be collected such as all-cause mortality, CVD mortality, myocardial infarction, coronary revascularization, stroke, thromboembolism, and all-cause hospitalization until week 52

### Participant timeline {13}

Enrolled participants will visit the site at 12, 26, and 52 weeks. The trial medication will be topped up at week 12 to last until 26 weeks after enrolment. A window period of ± 2 weeks will be permitted for the hospital visit. However, if participants were to come outside the window period, assessments would be done and assigned to the closest visit. The schedule of assessment is presented in Table 2.

**Table 2:** Schedule of assessment

Data collection	Screening	0 weeks	12 weeks	26 weeks	52 weeks
Eligibility assessment	X				
Pregnancy test^a^	X				
Informed consent and randomization		X			
Medications					
Trial medication compliance			X	X	
Concomitant medications	X				
Adverse effects			X	X	X
Assessments					
Resting blood pressure and oxygen saturation		X	X	X	X
NYHA class		X	X	X	X
6-min walk test (6MWT)		X	X	X	X
Dyspnoea score (Borg scale)		X	X	X	X
WBC count	X		X	X	X
C-reactive protein	X		X	X	X
Concomitant medications		X			
Lung function test					
Spirometry		X		X	X
Patient-reported outcome measures^b^					
EQ-5D-5L		X	X	X	X
Generalized Anxiety Disorder-7		X	X	X	X
Patient Health Questionnaire-9		X	X	X	X
Chalder Fatigue scale		X	X	X	X
Serious adverse events					
Sub-study population^c^					
High sensitivity troponin T		X		X	
NT-pro BNP		X		X	
Cardiac MRI		X		X	

^a^Women in reproductive age group (18–50 years), meeting all the other inclusion criteria and meeting no other exclusion criteria

^b^Translated questionnaires in local language

^c^Only for the Cardiac MRI sub-study

### Sample size {14}

A minimally clinically important difference of 30 m in 6MWT [21] was assumed for sample size calculation. We will recruit 350 participants to detect this difference, assuming a drop-out of 15% and a common standard deviation of 80 m (90% power at a two-sided significance level of 0.05). Out of these, 50 patients from each group will be randomly selected for the cardiac MRI sub-study.

### Recruitment {15}

The study participants will be screened from medical out-patient departments and COVID-19 survivors’ clinics. Patients in the COVID-19 hospital line listings will be telephonically invited for screening. Posters and audiovisual recordings about the study will be displayed within the hospital to motivate patients and hospital staff to volunteer. The recruitment from community centre is based on the house-to-house visits across the 67 villages in the Uddanam area. Trained field staff will screen individuals for their functional status based on the PCFS scale, specifically grade 2 or higher and check their CRP after recovering from the illness. These patients will be referred to the community health centre as part of a comprehensive care approach.

## Assignment of interventions: allocation

### Sequence generation {16a}

Randomization sequence (1:1 allocation ratio) will be generated (Stata17, RALLOC package) using varying block randomization stratified by site, hospitalization status at the time of acute infection, and body weight (≤ 70/ > 70 kg) at the time of randomization, by a researcher not involved in the day-to-day activities of this project at the sponsor institute.

### Concealment mechanism {16b}

Allocation concealment will be achieved using an interactive web-based randomization system integrated with REDCap (Research Electronic Data Capture) [22], hosted at the trial coordinating centre, which is a secure, web-based software platform designed to support data capture with audit trails, electronic signature features, inbuilt query management, and seamless data downloads to common statistical packages managed centrally by the data management team. Pre-numbered medication kits will be labelled as per the randomization sequence by two project coordinators external to the trial and couriered to the clinical sites.

### Implementation {16c}

At the clinical site, the site coordinator would enter the information of the stratifying factors of eligible consenting patients, following which the medication kit number will be revealed. The site and the trial coordinating centre will receive email notifications with the allocated kit number in real-time. The person enrolling the participant and dispensing the medication will not be aware of the kit number to be allocated until randomization. The medication bottles will be dispensed to the participants at baseline and week 12 visit.

## Assignment of interventions: blinding

### Who will be blinded {17a}

This is a double-blind placebo-controlled study where neither the participant nor clinician or the research team at the clinical site involved in participant recruitment, follow-up, and measurement of outcomes will be aware of the allocated treatment. The research staff at the trial coordinating centre involved in the day-to-day management of the trial and query management will not be not aware of treatment allocation. Only the unblinded data manager, statistician, and medication labelling team will have access to the randomization sequence and kit numbers. However, these personnel will not be involved in data or trial monitoring activities.

### Procedure for unblinding if needed {17b}

Only exceptional circumstances requiring knowledge of the actual treatment, essential for further management of the participant, would call for unblinding. This could be done by contacting an emergency trial helpline manned by a designated research staff at the trial coordinating centre not involved in the day-to-day management of the trial. The allocation will be sent by email to the trial investigator. An email stating that an unblinding has taken place will be sent to the coordination team and co-investigators for oversight purposes.

## Data collection and management

### Plans for assessment and collection of outcomes {18a}

All participants will be allotted a unique study identifier. Study data collected at the recruiting sites will be entered in electronic case report forms (eCRFs) by trained site staff according to the procedures documented in the eCRF manual into the password-protected trial-specific validated database with inbuilt checks to minimize data entry errors. Any post-entry queries will be resolved using the inbuilt query management system. Patient-reported outcome measures (PROMS) will be collected in the regional language by the research staff in paper and later entered in the trial database.

### Plans to promote participant retention and complete follow-up {18b}

The trial participants will be contacted once a month by the site research coordinator telephonically to build rapport and counsel them on the importance of compliance with the trial medication and maintaining the assigned visit schedule. For participants enrolled in the community outreach centre, the trial coordinator will visit the households in person. A participant newsletter in local languages will be developed to provide trial participants with an update on the trial progress. The trial-specific visits to the hospital will be aligned with routine clinical follow-up whenever possible. If the participant still cannot make it to the hospital for the follow-up visit, data (other than performance-based test) will be collected by a telephonic interview.

### Data management {19}

All collected data will be transferred securely to the database by the trained research staff. Several measures will be adopted to ensure data quality. These include inbuilt checks within the database to minimize data entry errors and ensure completeness and verification of critical data such as eligibility, lab reports, spirometry, and PROMs by a central trial monitor remotely via video conference. We will have a multi-pronged approach to ensure data quality instead of depending only on source data verification, which include inbuilt edit checks in the database, post-entry data routines and central data monitoring to identify abnormal data patterns in a periodic manner, and use of Power BI dashboards for the monitoring more frequently. Repeat training and feedback will be provided to the site coordinators to ensure data accuracy and completeness. Database will be locked after final quality checks and before unblinding. The final data will be handed over to the statisticians for unblinding and final analysis.

### Confidentiality {27}

Personal information, such as residential address and telephone number, will be collected by the site coordinator to enable follow-up visits. This information will not be entered in the trial database and will not be available to the central trial management team. The trial data will be linked only to the participant’s unique number identifier and not to any personal identifying information. Only clinical staff responsible for recruitment and follow-up can link trial data with participant personal identity. Any hospital records, if stored electronically at the trial site, will be anonymized before sharing for remote monitoring. Participant information such as consent forms, PROMs, laboratory reports, and other administrative forms will be housed in lockable file cabinets at the recruiting sites, with restricted access.

### Plans for collection, laboratory evaluation, and storage of biological specimens for genetic or molecular analysis in this trial/future use {33}

Not applicable since no biological sample collected for the genetic or molecular analysis.

## Statistical methods

### Statistical methods for primary and secondary outcomes {20a}

Primary and secondary effectiveness outcomes will be analysed using repeated measures mixed effects model. The main analysis will have changes from baseline values at 12, 26, and 52 weeks as the dependent variable. It will include the following as fixed effects: baseline value, treatment arm, visit time (26 and 52 weeks vs. 12 weeks), interaction between treatment arm and visit. A random site effect will be included to model within-site correlations, assuming an exchangeable correlation structure. The correlation between repeated measurements from the same participant will be modelled using an unstructured covariance matrix. The effect of the intervention will be estimated as the adjusted mean difference at 52 weeks, together with its 95% confidence interval. The same model will be used to estimate the effect at 12 and 26 weeks. This analysis will include all subjects with a baseline measurement and at least one post-baseline measurement. A detailed statistical analysis plan will be developed and published before unblinding and database lock.

### Interim analyses {21b}

Interim monitoring of blinded overall data will be presented 90 days after all sites have been initiated or when 50 patients have completed the 12-week follow-up.

### Methods for additional analyses (e.g. subgroup analyses) {20b}

Subgroup analyses will be defined based on patient-level characteristics (sex, hospitalization status, body weight (≤ 70 kg and > 70 kg), and CRP levels (> 2 and < 2 mg/dl)).

### Methods in analysis to handle protocol non-adherence and any statistical methods to handle missing data {20c}

All analyses will be conducted using a modified intention-to-treat population that includes all randomized patients eligible as per the protocol and who have taken at least one dose of the trial medication. The intention will be to include all subjects; however, some might be excluded from analyses due to missing data and may be subject to sensitivity analyses, e.g., imputations.

### Plans to give access to the full protocol, participant-level data, and statistical code {31c}

Anonymized trial data will be stored at the sponsor institute and can be made available upon reasonable request after the publication of the main results.

## Oversight and monitoring

### Composition of the coordinating centre and trial steering committee {5d}

The trial management committee is composed of co-investigators, trial manager, a site manager, and trial monitor and chaired by the chief investigator. It provides oversight of day-to-day management of the trial and prepares reports for the trial steering committee and data safety monitoring committee.

The steering committee comprises site investigators, external experts, a patient representative with lived experience due to persistent symptoms after COVID-19 infection and is chaired by the chief investigator. The committee will be responsible for reviewing trial progress periodically in terms of recruitment, retention, safety reporting, and approving any protocol amendments. At the time of writing this manuscript, the committee had met twice.

### Composition of the data monitoring committee, its role and reporting structure {21a}

A four-member independent data safety monitoring committee (DSMC) includes a statistician not involved in random sequence generation to develop the blinded interim reports that will help DSMC to assess trial progress, medication compliance, safety of trial participants, and data quality. Unblinded reports will be presented if only required by the DSMC. Details of the DSMC roles and responsibilities, meeting format, and reporting structure are elaborated in the DSMC charter (supplementary). Recommendations to amend the protocol or change the conduct of the trial made by the DMSC will be considered by the steering committee. The steering committee has exclusives rights for deciding whether to continue, hold, or stop the trial based on the DMSC recommendations.

### Adverse event reporting and harms {22}

At therapeutic doses of colchicine, apart from (mild and transient) gastrointestinal disturbances, no concerning major adverse effects have been reported in literature. A predefined list of adverse symptoms (e.g. diarrhoea, nausea, vomiting, abdominal pain, sore throat) will be asked at every visit. Any other adverse effects reported by the participants will be recorded in the CRFs. Participants will be counselled to contact the site research coordinator immediately in case of any adverse symptoms.

All serious adverse events will be treated at the recruiting hospital, documented in trial-specific forms, and reported to the trial sponsor within 24 h of occurrence or knowledge of the event. The site principal investigator and research staff will document the time of onset, duration, resolution, and actions taken as well as an assessment of the intensity and relationship of the event with the study treatment. This complete report will be submitted to the local ethics committee and to the sponsor for a complete investigation of causality within 14 days.

### Frequency and plans for auditing trial conduct {23}

No external or internal audit is planned for this trial except for routine trial monitoring. The PROMs, hospital records, and all original signed informed consent forms made available by the clinical investigator will serve as a source for data verification by the trial monitor at the trial coordinating centre during remote or on-site trial monitoring. The query management system to raise, respond, and close the query with changes to the responses will be recorded with an audit trail.

### Plans for communicating important protocol amendments to relevant parties (e.g. trial participants, ethical committees) {25}

Any protocol amendments made during the study will be approved by the ethics committee before the implementation and updated in the trial registry.

There was one protocol amendment related to inclusion criteria. When the trial was initiated, those who were more than 100 days (3 months) after COVID-19 infection were excluded even if they had persistent symptoms. The amended inclusion criteria removed the upper limit of days since COVID-19 infection and were approved by the trial steering committee. The modified protocol was approved by the ethics committee of the sponsor and all participating centres.

### Dissemination plans {31a}

The trial results will be published in a peer-reviewed biomedical journal irrespective of the direction of the result as per the Consolidated Standards of Reporting Trials (CONSORT) statement. All members of the protocol writing group and the site investigators will be co-authors in the manuscript. Any other researcher meeting the ICMJE authorship criteria will also be granted co-authorship. Site-specific data or sub-study results or any other exploratory analyses will not be analysed until the publication of the main result. The steering committee will approve the manuscripts from this trial before the decision to publish. The funding agency will be acknowledged, but they will have no influence on the data analyses, interpretation of trial results, writing of the manuscript, or the decision to publish. The study results will be shared with the trial participants via a newsletter.

## Discussion

There is a compelling need to find an effective preventive and therapeutic strategy to reduce the burden of long COVID symptoms. The burden is likely to be greater due to large number of undocumented cases and multiple possible adverse outcomes and effect on quality of life. There is emerging real world evidence on Nirmatrelvir (Paxlovid) [23] and mRNA covid vaccine [24] in preventing long COVID. However, conclusive evidence from randomized controlled trials [25] is yet to be generated for prevention and treatment of long COVID.

This trial addresses an important health priority and can rapidly translate research into best practice and policy. This is a placebo-controlled randomized trial with effective blinding procedures in place. We will collect both subjective and objective outcomes. Standardized protocols will be used to minimize error for performance-based outcomes such as 6MWT and spirometry-based lung volumes. We will engage the community by organizing a webinar to understand burden of symptoms from COVID-19 survivors. We have a patient representative in the steering committee, and we will periodically thank trial participants via newsletters that includes a lay person summary of trial recruitment and follow-up status.

## Limitation

When we initiated the trial, a lot is unknown including a standard definition and symptomatology of long COVID. There are still no clearly accepted diagnostic tests or biomarkers to detect and monitor the disease. Based on existing literature on pathophysiology, we used functional limitation along with elevated inflammatory markers as a criterion to enrol participants. Emerging definitions also led to changing the inclusion criteria of the trial after some months into the trial. The participating trial sites are heterogenous, which could increase the generalizability of the trial findings but could increase noise to the outcome data in a statistical perspective.

## Trial status

At the time of writing the manuscript, the trial is ongoing.

Protocol version number: 2.0

First day of recruitment: February 11, 2022.

Protocol version number: 2 (April 12, 2022).

Last patient recruited: July 21, 2023.

Expected last patient last follow-up: July 2024. We aimed to submit the protocol before the end of participant recruitment; however, there was a delay in obtaining feedback from all the co-authors and in submitting the manuscript.

## Data Availability

Only researchers with ethical approval will have access to the final datasets after the completion of the study. The datasets analysed will be available from the corresponding author upon reasonable request.

## Abbreviations

6MWT: 6-Min walk test
ACE2: Angiotensin-converting enzyme 2
COVID-19: Coronavirus disease
HS-CRP: High-sensitive C-reactive protein
IL: Interleukin
NLRP3: Nucleotide-binding oligomerization domain-, leucine-rich repeat-, and pyrin domain-containing protein 3
PCFS: Post-COVID-19 functional status
PROMS: Patient-reported outcome measures
SARS-CoV-2: Severe acute respiratory syndrome coronavirus 2
